# Synergism of Dietary Co-Supplementation with Lutein and Bile Salts Improved the Growth Performance, Carotenoid Content, Antioxidant Capacity, Lipid Metabolism, and Lipase Activity of the Marbled Spinefoot Rabbitfish, *Siganus rivulatus*

**DOI:** 10.3390/ani10091643

**Published:** 2020-09-12

**Authors:** Abdallah Tageldein Mansour, Mohamed M. M. El-feky, Hossam S. El-Beltagi, Ahmed Elsayed Sallam

**Affiliations:** 1 Animal and fish Production Department, College of Agricultural and Food Sciences, King Faisal University, P.O. Box 420, Al-Ahsa 31982, Saudi Arabia; 2 Fish and Animal Production Department, Faculty of Agriculture (Saba Basha), Alexandria University, Alexandria 21531, Egypt; 3 National Institute of Oceanography and Fisheries (NIOF), Cairo 21624, Egypt; dive_mmae2010@yahoo.com; 4 Agricultural Biotechnology Department, College of Agriculture and Food Sciences, King Faisal University, P.O. Box 420, Al-Ahsa 31982, Saudi Arabia; helbeltagi@kfu.edu.sa; 5 Biochemistry Department, Faculty of Agriculture, Cairo University, Gamma St.Giza 12613, Egypt

**Keywords:** bile salts, lutein, carotenoid content, antioxidant, growth, rabbitfish (*Siganus rivulatus*)

## Abstract

**Simple Summary:**

In the natural aquatic environment, fish obtain their carotenoid requirements from the natural food web. Meanwhile, under aquaculture conditions, especially intensive culture, fish are deprived in terms of their carotenoids requirements from the environment. Accordingly, the artificial diet formula must consider the carotenoids accessibility and maintain its efficient utilization. Moreover, in fish larvae, the absorption of carotenoids as lutein (LTN) is not efficient. Therefore, as LTN is fat-soluble carotenoid, the improvement of fat digestion and absorption could improve LTN utilization. The present work evaluated the effect of individual or co-supplementation of LTN and bile salts (BS) in the diets of rabbitfish (*Siganus rivulatus*) larvae. The results revealed an improvement of growth performance and survival (%) with dietary supplementation with LTN and BS, which could enhance the cost–benefit of farming rabbitfish. Also, the carotenoid deposition, antioxidant status, lipase activity, and lipid metabolism improved with co-supplementation of LTN and BS than its individual supplementation.

**Abstract:**

A 60-day feeding trial was conducted to assess the effects of dietary supplementation with bile salts (BS), lutein (LTN), and their combination on growth, survival, carotenoid content, and antioxidant status of rabbitfish (*Siganus rivulatus*) larvae. Fish were fed four isonitrogenous (34.39% protein) and isoenergetic (20.57 kJ/g) diets supplemented with BS (0.15 g kg^−1^), LTN (0.1 g kg^−1^), BS+LTN (0.15 and 0.1 g kg^−1^, respectively), and a non-supplemented control diet. The results revealed that fish fed BS+LTN had the highest significant specific growth rate (4.37% day^−1^), feed efficiency (46.55%), and survival (97.78%). Lutein supplementation improved whole-body protein content, meanwhile, fish fed a BS-supplemented diet had a higher lipid content. The carotenoid deposition was significantly increased with LTN and BS+LTN in skin, muscle, and whole body compared to the control and BS treatment. All dietary supplementation of BS and LTN showed significant improvement in total antioxidant capacity, catalase, and glutathione peroxidase activities. Additionally, LTN alone or BS+LTN significantly reduced malondialdehyde levels by 5.30 and 29.91%, respectively compared to the control. BS supplementation modulated aminopeptidases activities, triglycerides, cholesterol, and increased the activity of pancreatic lipase. Therefore, it could be inferred that dietary supplementation with LTN in combination with BS could improve the growth performance, carotenoid deposition, antioxidant status, lipid digestion, and metabolism of *S. rivulatus*.

## 1. Introduction

Global aquaculture production has been on a continuous rise to cover the increase in the global consumption of aquatic animals due to rapid population growth and consumer awareness of the decline/deterioration of natural fisheries [1]. Presently, the marketing of aquatic products, considering the nutritive values, especially antioxidant levels, fatty acid profile, and trace element content, highlights their use as functional foods [2,3,4,5]. Carotenoids are one of the preferred supplements, as a natural pigment, to be delivered via aquatic products to improve general body performance and some specific functions, including ocular integrity [6,7]. That is in addition to its positive effects on the farmed animal itself, including high growth, survival, immunity, and stress relief [2,8,9,10].

Carotenoids are the most widespread lipophilic pigment in photosynthetic organisms [11]. These tetraterpene compounds are divided into two groups: carotenes, including α-β-carotenes and lycopene, and xanthophylls, including lutein (LTN) and zeaxanthin [12]. These compounds are light-harvesting pigments in the photosynthesis process and have several biological functions in the live body, mainly due to free radical scavenging capacity [2,11,13]. The marketing of carotenoids has seen an increasing annual growth rate (5.7%) and LTN, as one of main commercial carotenoids, has represented 23% of carotenoids marketing with high growth potential [14]. Lutein is an oxygenated derivative of carotenoids as a part of the xanthophyll family [12]. Moreover, LTN is found in several plants and micro-organisms and the main commercial source of LTN is marigold flower, *Tagetes sp,* followed by microalgae, such as *Scenedesmus almeriensis*, *Tetracysis aplanosporum,* and *Desmodesmus protuberans* [11,15,16]. Purified LTN (yellow-orange crystalline) is classified as generally recognized as safe to be used as a feed supplement [17]. Lutein has higher antioxidant activity than *β*-carotene [18] and in addition, LTN has anti-atherosclerotic [19], anti-inflammatory [20], and anti-hyperlipidemic effects [21].

In the natural aquatic environment, fish obtain their carotenoid requirements from the natural food web, including aquatic plants, algae, and micro-organisms, which reflects its coloration and brightness and participates in maintaining normal animal performance [22,23]. Therefore, under the conditions of fish farming, an artificial diet formula must be considered for carotenoid accessibility because fish do not have the ability to synthesis it and a deficiency of carotenoids affects pigmentation, immunity, and the antioxidant system [23,24,25]. LTN bioavailability is critically affected by its absorption rate [12]. LTN esters require prior de-esterification by intestinal hydrolysis enzymes before being transported in the bloodstream via both low- and high-density lipoprotein cholesterol [17]. Therefore, improving lipid digestibility and absorption could improve carotenoid and LTN utilization and assure a cost-effective diet formulation [2,12].

Bile salts (BS) are conjugated bile acids with glycine or taurine secreted by hepatocytes in response to dietary lipids. They have an emulsification effect on lipids to facilitate the enzymatic digestion and absorption of fat and fat-soluble nutrients (vitamins, carotenoids, and phospholipids) [26]. Furthermore, BS also have several metabolic functions, such as the activation of bile salt-activated lipase in fish [27], improving nutrient absorption [28], increasing the solubility of amino acids (glycine and taurine) [29], and maintaining cholesterol homeostasis in the liver [26,29], among others. 

Dietary supplementation with BS improved antioxidant systems, liver function, and histological structure and had an anti-inflammatory effect in juvenile black sea bream, *Acanthopagrus schlegelii* [30]. In addition, dietary supplementation with BS improved growth performance, feed utilization, and antioxidant systems of European seabass, *Dicentrarchus labrax* [2], grass carp, *Ctenopharyngodon idella* [31], and Nile tilapia, *Oreochromis niloticus* [32]. BS supplementation in a soybean meal-based diet improved the growth performance and feed utilization in rainbow trout, *Oncorhynchus mykiss* [33].

Siganids (rabbitfish) are a small family of marine algaevorous fish that are widely distributed in the Red sea and Indo-West Pacific region; these species invaded the eastern Mediterranean sea after Suez canal construction [34,35]. Among the rabbitfish species, the Marbled spine foot rabbitfish, *Siganus rivulatus,* is a potent candidate for marine aquaculture diversification in cages, offshore ponds, and recirculated aquaculture systems [36,37,38]. This species holds particular promise for aquaculture because of their herbivorous feeding habits, fast growth rate and high tolerance to ammonia, disturbances in environmental conditions, handling stress, and high stocking density [39,40]. Additionally, rabbitfish have a high market value in Egypt, Jordan, Saudi Arabia, and other countries in the Middle East and around the Mediterranean basin [39,41]. In addition, in captivity, rabbitfish can be readily trained to accept artificial vegetarian or low fish meal diets, making them suitable for commercial aquaculture, but it could miss its natural pigmentation [42]. However, studies on rabbit fish best aquaculture practices and feeding regime are still inadequate to introduce knowledge for the farmers to maximize growth, feed utilization, physiological status, and body color as a market merit of this species [39,43,44]. Therefore, the present study aimed to evaluate the effects of dietary supplementation with BS and LTN, individually or in combination, on growth performance, body composition, total carotenoid content, antioxidant status, liver function, and lipase activity of rabbitfish, *S. rivulatus,* larvae.

## 2. Materials and Methods

### 2.1. Fish and Culture Condition 

Marbled spinefoot rabbitfish, *S. rivulatus,* were collected from the coastal waters of Alexandria governorate, and immediately transported to El-Max Research Station, National Institute of Oceanography and Fisheries (NIOF) (Alexandria, Egypt). Prior to the experiment, all fish were reared in cages and fed the control diet for 4-weeks to acclimatize to the experimental conditions and equalize their body carotenoids content. 

At the start of the feeding experiment, the fish were fasted for 24 h before weighing. A total of 600 healthy fish of equal size (mean weight: 0.18 ± 0.02 g and mean length: 2.52 ± 0.17 cm) were selected and allotted randomly into twelve experimental hapa cages (100 × 100 × 70 cm; 50 fish per cage) (three replicates/cages group^−1^). Each group was installed in one outdoor rectangular concrete tank (6 × 2 × 1 m; 12 m^3^ water capacity). Fish were hand-fed on their prescribed diet three times a day (9:00, 12:00 and 15:00 h) slowly to apparent satiation (achieved by allowing fish to eat until feeding activity stopped, with no feed remaining in the bottom of the cage, and the amount of feed being consumed by the fish from each cage were recorded) for a period of 60 days. All experimental protocols were approved by Institutional Animal Care and Use Committee of Alexandria University (IAUC) with the approval No. AU: 14200721311.

During the experimental period, the concrete tank continuously aerated using air stones diffusers to maintain the dissolved oxygen level above 5 mg L^−1^ and about 30% of water was replaced daily with filtered saltwater (salinity 32 ppt). Water temperature was 26.0 ± 1.0 °C, pH 7.5–8.2, NH_3_-N < 0.2 mg L^−1^ (digital multi-meter; Crison, model MM41, Barcelona, Spain). The photoperiod followed the natural cycle (light: dark 12:12 h).

### 2.2. Experimental Diets Formulation 

A total of four experimental diets were formulated to be isonitrogenous (crude protein: 34.39%), isolipidic (crude lipid: 9.81%) and isocaloric (gross energy: 20.57 kJ g^−1^) as pre-determined requirements of *S. rivulatus* according to Ghanawi, et al. [44]; El-Dakar, et al. [45] and Abou-Daoud, et al. [46], and shown in Table 1. The control diet was prepared without LTN or BS supplementation (Control), and the other three diets were control diet supplemented with BS (0.15 g kg^−1^; Handan Qinyueming Metal Products Co. Ltd., Hebei, China), LTN (0.1 g kg^−1^; Jeevan Chemicals and Pharmaceuticals Co., Maharashtra, India) and combination of BS+LTN (0.15 and 0.1 g kg^−1^, respectively). The inclusion level of LTN and BS in this study were selected according to the previous studies of [25] and [32], respectively. 

Briefly, all dried raw materials were shattered in a blender, sieved into <200 μm particle size before being weighed accurately (~0.1 g). Sunflower oil and then warm distilled water (about 30% of total dry mixture) were added to produce dough. The stiff dough was then pelleted (1.0 mm diameter) with a meat grinder machine (Tornado MG−2000, Egypt). The feed pellets were dried in a ventilated oven at 40–50 °C for approximately 5 h and then allowed to cool overnight at room temperature. The dry diets were labelled according to the treatments and stored in plastic-lined bags at −20 °C until usage.

### 2.3. Samples Collection

#### 2.3.1. Fish Sampling

Before starting the growth trial, 30 fish were randomly collected to measure the initial body weight and total length. At the end of feeding trial, fish were starved for 24 h from the last feeding, weighed, and counted to determine growth performance parameters, feed utilization, and survival rate in each treatment. Also, around 20 specimens were randomly selected from each cage, stored at –20 °C for whole-body composition analysis. The fish samples were collected in the morning for all sampling times.

#### 2.3.2. Tissues Sampling

Randomly three fish per cage were dissected after killing with overdose of clove oil (5 mg L^−1^) and used to analyze total carotenoids in skin and muscle, where skin was taken from the dorso-lateral part and muscle tissue was taken from the base of the dorsal fin of the fish. Also, other three whole fish were collected to determine total carotenoid in the whole-body. The samples were kept in dark plastic bags and stored at −20 °C until further analysis. In addition, to determine antioxidant status and some biochemical parameters, liver samples of the dissected fish were collected, rinsed with cold saline and homogenized in ice-cold buffer solutions (50 mM potassium phosphate, 1 mM EDTA; pH 7.5.) using a Wise Tis^®^ HG−15D homogenizer (Daihan Scientific, Bangalore, India) to prepare (1:9 *w*/*v*) homogenates. The homogenates were centrifuged at 750× *g* for 15 min at 4 °C, and the resulting supernatants were immediately stored at −80 °C until determined the enzyme activities.

### 2.4. Measured Parameters

#### 2.4.1. Growth Index and Feed Efficiency 

The recorded initial, and final body weight (FBW), initial and final body length (FBL; total length) and feed intake were used to calculate the following growth and feed efficiency indexes:

Weight gain (WG; g) = final weight (g) − initial weight (g)(1)

Specific growth rate (SGR; % day^−1^) = [ln (final body weight) − ln (initial body weight)]/days (d) ×100 (2)

Body length growth rate (BLGR; % day^−1^) = [ln (final body length) − ln (initial body length)]/days (d) ×100 (3)

Feed efficiency (FE; %) = 100 × weight gain (g)/feed intake (g)(4)

Feeding rate (FR; % BW day^−1^) = 100 × feed intake/[days of the experiment × (initial body weight + final body weight)/2] (5)

Survival (SR; %) = 100 × final fish number/initial fish number(6)

Condition factor (CF) = 100 × body weight (g)/body length (cm)^3^ (7)

#### 2.4.2. Proximate Chemical Analysis

Proximate analysis of experimental diets and whole body of fish were analyzed in triplicate according to methods of Association of Official Analytical Chemists [48]. In particular, moisture content was determined after samples were dried in an oven at 105 °C for more than 12 h. Protein content was determined by measuring total nitrogen (N × 6.25) levels using the Kjeldahl method following acid digestion with an Auto Kjeldahl System (K358, BUCHI, Flawil, Switzerland). Fat content was detected by ether extraction using a Soxhlet System (Model VELP Scientifica, SER 148, Italy) and ash content was determined by muffle furnace at 550 °C for 5 h.

#### 2.4.3. Analysis of Total Carotenoids in Fish and Diets

Total carotenoids were analyzed in fish and diets using the method described by Olson [49]. Briefly, samples of skin, muscle, and whole body (0.25 g) and feed (1 g) were gently mashed with 2.5 g of anhydrous sodium sulphate in glass vial. Then 5 mL of chloroform was added to the sample and left overnight at 0 °C, until a clear layer above the caked residue was formed. About 0.3 mL of chloroform diluted to 3 mL with absolute ethanol. The optical density of the sample was measured by a spectrophotometer (PD-303 UV, APEL, Kawaguchi, Japan) at 380, 450, 470, and 500 nm. The wavelength at which maximum absorption, was used for the calculation:

Total carotenoid content = (10 × absorption at max. wavelength)/[0.25 × sample weight (g)]

#### 2.4.4. Determination of Antioxidant Enzymes

Malondialdehyde (MDA) was measured as Thiobarbituric acid (TBA) according to Draper et al. [50]. TBA reacts with MDA in acidic medium at temperature of 95 °C for 30 min to form thiobarbituric acid reactive product the absorbance of the resultant pink product can be measured at 534 nm. An extinction coefficient of 156,000 M^−1^ Cm^−1^ was used for the calculation. 

Total antioxidant capacity (T-AOC) was estimated using the method of Cao, et al. [51]. Briefly, the determination of the antioxidative capacity is performed by the reaction of antioxidants in 0.02 mL of homogenates with 0.5 mL of hydrogen peroxide (H_2_O_2_) buffer, incubated for 10 min at 37 °C. The antioxidants in the sample eliminate a certain amount of the provided hydrogen peroxide. The residual H_2_O_2_ is determined colorimetrically by an enzymatic reaction which involves the conversion of 3,5-dichloro-2-hydroxyl benzensulphonate to a colored product assessed at 505 nm. 

Activity of hepatic Glutathione peroxidase (GSH-Px) was measured according to the method of Noguchi, et al. [52]. One unit of GPx activity was defined as the amount of enzyme that reduced the glutathione (GSH) concentration in the reaction system at 1 μmol/L per min.

Catalase activity (CAT) was assayed according to Sinah [53], in which dichromate in acetic acid is reduced to chromic acetate in the presence of H_2_O_2_ when heated, forming perchromic acid as an unstable intermediate. The reaction mixture contained 100 μL of liver samples, 500 μL of peroxide hydrogen, and after the reaction dichromate/acetic acid reagent (3:1) was used. The absorbance reading was performed at 610 nm.

Aspartate aminotransferase (AST) and alanine aminotransferase (ALT) were assayed as described by Wootton (1964) with 0.2 M DL-aspartic acid and 20 mM L-ketoglutarate as the substrate and 0.2 M DL-alanine and 2 mM L-ketoglutarate, respectively.

Triglyceride (TG) was analyzed in liver using the triglyceride quantification Kit (MAK266, Sigma-Aldrich, St Louis, MO, USA) [54]. In this assay, TG are converted to free fatty acids and glycerol. The glycerol is then oxidized to generate a colorimetric product and the absorbance was read at 570 nm., and total cholesterol, were estimated using the Cholesterol Quantitation Kit (MAK043, Sigma-Aldrich, St Louis, MO, USA) [55]. In this kit, total cholesterol concentration is determined by free cholesterol and cholesteryl esters enzyme assay, which results in a colorimetric product and the absorbance was read at 570 nm. 

Lipase activity in liver tissues was estimated using the lipase activity assay kit (Cayman Chemicals, Ann Arbor, MI, USA). Lipase activity was determined using a coupled enzyme reaction, which results in a colorimetric (570 nm) product proportional to the enzymatic activity present. One unit of Lipase is the amount of enzyme that will generate 1.0 µmole of glycerol from triglycerides per minute at 37 °C.

### 2.5. Statistical Analysis

The results are presented as mean ± SE of three replicates. The normal distribution and homogeneity of the data were confirmed before the statistical analysis. All data were statistically analyzed as a completely randomized design by ANOVA using SPSS (Standard version 17.0; SPSS, Chicago, IL, USA). Tukey test was used to compare the differences between means when significant F values were observed at the *p* ≤ 0.05 levels.

## 3. Results

### 3.1. Growth and Feed Efficiency

The growth performance and feed efficiency parameters of rabbitfish, *S. rivulatus,* fed BS, LTN, and BS+LTN for 60-days feeding period are presented in Table 2. There was a significant increase in FBW, FBL, WG, SGR, BLGR, and FE of fish fed BS+LTN supplemented diet compared with other treatments. The feeding rate was significantly decreased in fish fed the BS+LTN supplemented diet compared to other groups. The condition factor did not significantly affect in the studied groups and ranged from 1.19 to 1.26. In addition, the survival (%) ranged from 86.67% to 97.78% and the highest survival was obtained in fish fed the BS+LTN diet with a significant increase compared to the control group.

### 3.2. Whole-Body Proximate Composition

The whole-body proximate chemical composition data of the different experimental groups are presented in Table 3. The moisture content decreased significantly in the BS+LTN group compared to other treatments. Crude protein increased significantly in groups fed LTN supplemented diets (LTN and BS+LTN) compared to other treatments. Meanwhile, crude lipid increased significantly in BS supplemented groups (BS and BS+LTN). Ash contents did not show any significant differences among all treatments.

### 3.3. Carotenoid Content in Fish and Experimental Diets.

Lutein supplementation increased the total carotenoid content significantly compared to the control and BS supplemented diet (Table 4). In addition, the whole-body, skin, and muscle contents of carotenoids increased significantly in groups fed LTN alone or in combination with BS supplemented diets compared to other groups (Table 4). The carotenoid content tended to improve with BS supplemented diet.

### 3.4. Antioxidant Enzyme Activities

**Figure 1:**
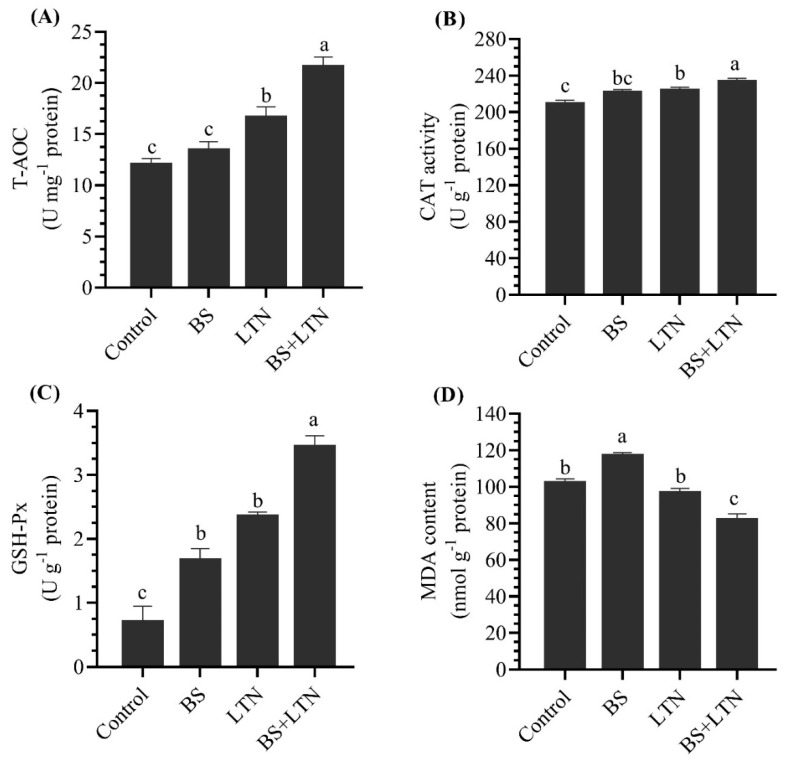
Effects of dietary supplementation with lutein (LTN) and bile salts (BS) on total antioxidant capacity (**A**), catalase activity (**B**), glutathione peroxidase activity (**C**), and malondialdehyde content (**D**) of *Siganus rivulatus* liver homogenate after 60 days of feeding. All values are presented as mean ± SE (n = 3). Different letters above the bars denote significant differences among four groups at the *p* < 0.05.

Figure 1 shows the change in antioxidant enzymes activities and MDA levels in the hepatopancreas of *S. rivulatus* fed the experimental diets. The activities of T-AOC, GSH-Px, and CAT increased significantly with fish fed BS, LTN, and their combination compared to fish fed the control group. The highest level of antioxidant enzymes activities was recorded with fish fed a BS+LTN supplemented diet. Dietary supplementation with BS+LTN significantly decreased the MDA content (*p* < 0.05) compared to other groups. 

### 3.5. Liver Function and Lipase Activity 

**Figure 2:**
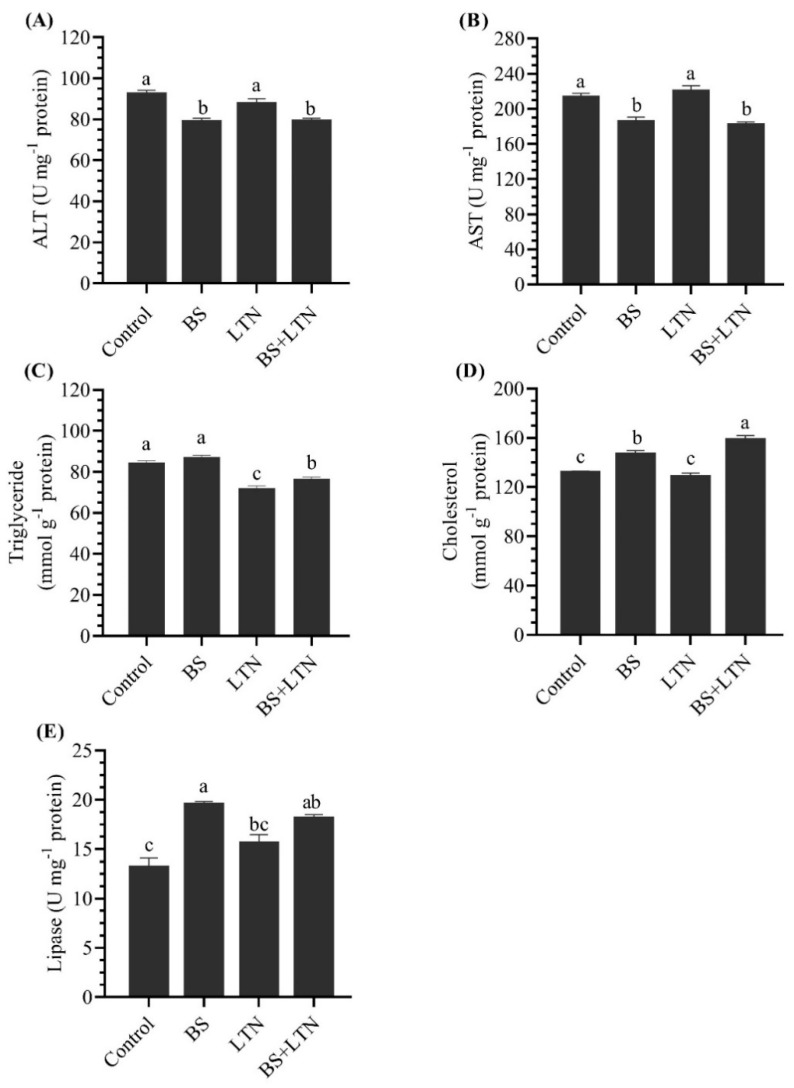
Effects of dietary supplementation with lutein (LTN) and bile salts (BS) on alanine aminotransferase (**A**), aspartate aminotransferase (**B**), triglyceride (**C**) cholesterol (**D**), and lipase (**E**) of *Siganus rivulatus* liver homogenate after 60 days of feeding. All values are presented as mean ± SE (*n* = 3). Different letters above the bars denote significant differences among four groups at the *p* < 0.05.

ALT and AST activity in the hepatopancreas were significantly decreased in BS and BS+LTN (*p* < 0.05) supplemented groups compared to the LTN and control group. In addition, the total cholesterol level in fish fed a BS+LTN supplemented diet was significantly higher than those found in both LTN and BS groups (*p* < 0.05), while the highest and lowest TG level was shown in the BS group and LTN group, respectively (Figure 2). 

There was high activity of the lipase enzyme in the hepatopancreas homogenate of fish that received the BS supplemented diet (19.53 ± 0.30), followed by the BS+LTN diet (18.12 ± 0.36) (Figure 2).

## 4. Discussion

The improvement of growth performance, feed utilization, and physiological status of cultured fish is a way to maximize economic revenue [4,56]. This target has a great impact on the establishment of farming emerging aquaculture species, such as rabbitfish, *S. rivulatus* [39]. One solution to fulfill this strategy is dietary supplementation, such as with carotenoids that have antioxidant activity and can improve growth performance, feed utilization, immune response, and reduce inflammation [2,8,9,10,57]. However, carotenoid absorption is not efficient in young fish [2,58]. Moreover, LTN is not as readily absorbed as other carotenoid sources (astaxanthin and canthaxanthin) [25,59]. Accordingly, the present study examined the effect of LTN and bile acids, individually or in combination, on the growth performance, feed utilization, body composition, carotenoid deposition, and antioxidant status of rabbitfish, *S. rivulatus*.

Dietary LTN supplementation alone did not affect growth performance (weight and length indices) and feed efficiency in the present study. Similarly, LTN supplementation did not significantly improve the growth of goldfish, *Carassius auratus* and Lake Kurumoi rainbow fish, *Melanotaenia parva* [23,25]. In addition, several studies indicated that feeding LTN or carotenoid sources did not exert any marked effects on growth performance and feed utilization of different fish species, including Atlantic salmon, *Salmo salar* [60], characins, *Hyphessobrycon callistus* [18], rainbow trout, *O. mykiss* [61,62], and Nile tilapia, *Oreochromis niloticus* [63]. These results could be attributed to the poor absorption of carotenoids in fish larvae [2,58,64]. Whereas, the larvae digestive enzymes activities and absorption still in a developmental stage during the metamorphosis [65]. Furthermore, LTN did not affect the digestibility coefficient of protein, lipids, and dry matter in fish and dogs fed an LTN supplemented diet [25,66].

Moreover, BS supplementation alone did not improve the growth and survival of rabbitfish in the present study. Accordingly, there were no significant differences in growth performance and feed utilization between fish fed a BS supplemented diet and the control [30]. However, grass carp, *C. Idella*, fed a diet supplemented with BS showed an improvement in growth performance, intestinal growth and functions, and gut microbiota homeostasis [67]. Additionally, rainbow trout, *O. mykiss*, fed a diet supplemented with BS attained efficient utilization of dietary protein and normal intestinal histology [68]. These discrepancies between our findings and others could be due to differences in the BS source and fish species, experimental condition, feed processing, and feeding duration.

Meanwhile, a remarkable increase in growth performance, feed efficiency, and survival (%) was observed in fish fed the combination of LTN and BS in the current findings. In accordance, black seabream, *A. schlegelii*, fed a combination of BS and L-carnitine recorded the highest growth performance and feed utilization compared with individual supplementations [30]. BS supplementation could improve growth performance by enhancing nutrient absorption pathways via regulating ionic transport in the intestine of Senegalese sole, *Solea senegalensis* [28]. Additionally, LTN has antioxidant activity and can modulate the antioxidant balance in fish [69,70], which was confirmed in the current findings (Figure 1). However, the effect of BS and LTN as individual supplementation was not clearly obtained in rabbitfish but co-supplementation elucidated a significant improvement in growth performance and feed utilization. 

The synergistic effect of BS and LTN could be attributed to the improvement of absorption and metabolism of fat and fat soluble components, such as LTN, whereas, BS has an amphipathic characteristic and is capable of transforming lipid bilayers to mixed micelles and facilitates the hydrolysis process induced by lipases [71,72,73]. Accordingly, the determination of carotenoid content in the present study showed the highest accumulation in the BS+LTN supplemented group (Table 4). Moreover, BS could maximize the role of lipids as a protein sparing effect, especially in fish, which use lipids as the main source of metabolic energy [28,67]. Additionally, this could be interpreted as the significant increase in the whole-body ether extract of fish fed BS alone or in combination with LTN in the current findings.

The whole-body protein content, as an indicator of fish nutritional quality, increased significantly in fish fed LTN alone or in combination with BS. In line with the results of this study, dietary supplementation of different carotenoid sources with or without taurocholate significantly increased the protein content in European seabass, *D. labrax* [2]. Additionally, dietary supplementation with *Spirulina platensis* and *Rubrivivax gelatinous* increased the protein content, modulated the fatty acids profile, and preserved the fillet quality of Nile tilapia [63]. 

Moreover, fish visual appearance (pigmentation, and species-specific Marble color) is a market preferred characteristic in table fish and ornamental fish, in addition to nutritional quality [39,43]. However, fish cannot synthesize their own coloring pigments *de novo*, thus, external sources should be provided [25,74]. Lutein is a yellow pigment responsible for yellowish–orange coloration [23]. In the present study, external pigmentation of rabbitfish skin revealed a marked yellow pigmentation and brightness in fish fed the BS+LTN supplemented diet. Additionally, the skin, muscle, and whole-body content of carotenoids in fish fed LTN in combination with BS was significantly higher than the control. The determination of skin carotenoids is an accurate indicator for the quantitative fish skin pigmentation [23], whereas the coloration parameters were linearly related to the carotenoid content in the skin [75]. In fish, the biotransformation and degradation of LTN via oxidative and reductive pathways is not completely understood and differ from species to other [76,77]. In contrast, dietary LTN was oxidized to canthaxanthin, zeaxanthin, and β−carotene in rainbow trout, masu salmon, and black roch fish, respectively, or it can be directly deposited as in eels [76].

In the same sense with the current findings, dietary LTN at a dose of 75 mg kg^−1^ of feed provided higher yellowness and carotenoid content in the skin of the large yellow Croaker, *Larimichthys croceus*, [75]). Dietary LTN in powdered and nano forms significantly increased LTN levels in plasma, liver, eyes, and adipose tissue of guinea pigs [78]. European seabass, *D. labrax*, fed different diets supplemented with synthetic astaxanthin, marigold flower meal, and crab meal as sources of carotenoids exhibited higher carotenoid content [2,13]. Furthermore, Goldfish, *C. auratus,* fed LTN supplemented diets showed a high carotenoid content [23]. In addition, increasing dietary astaxanthin and β-carotene increased the body content of astaxanthin in a dose dependent manner [18]. Lutein, β-carotene, and lycopene supplementation increased the total carotenoid in raw and processed Nile tilapia fillets and improved the fatty acid profile and fatty acids stability [5]. 

Bile acids supplementation positively improved carotenoid content in skin, muscle, and the whole body, and the synergistic effects of BS and LTN exhibited the highest carotenoid accumulation in the current findings. In accordance, taurocholic acid supplementation increased the blood astaxanthin level by 20% more than in the non-supplemented group [79]. Also, European seabass, *D. labrax*, fed different sources of carotenoids in combination with sodium taurocholate maximized the absorption, accumulation, and efficient effects of carotenoids [2].

The findings of the present study showed that high deposition of the carotenoid pigment was translated into an improvement in antioxidant status. Furthermore, dietary supplementation with LTN, BS, and their combination significantly improved T-AOC, GSH-Px, and CAT activities and reduced MDA levels in the liver homogenate of rabbitfish. In the same sense, LTN supplementation significantly increased plasma T-AOC and decreased malondialdehyde levels [69]. LTN reduced serum oxidative stress markers (TBARS and ROS) and improved glutathione levels, CAT, SOD, GSH-Px, and GST activities in plasma of ovariectomized rats [70]. Additionally, LTN supplementation improved the non-enzymatic antioxidants (total sulfhydryl and non-protein sulfhydryl groups) and reduced TBARS levels in the LTN supplemented groups [80,81,82].

Lutein could also modulate antioxidant gene expression (HO−1, NQO1) and inflammatory response genes (Nrf2 and decreases NF-kB genes) [70]. The antioxidant properties of LTN could be attributed to the presence of two hydroxyl groups in each molecule [83]. Therefore, LTN has the ability to quench the singlet oxygen and other ROS by transferring an electron to form a radical cation or by the addition of an electron to form an adduct [83,84]. In addition to quenching ROS directly, LTN was reported to effectively prevent protein, lipid, and DNA from oxidative damage by regulating other cellular antioxidant systems [82]. Whereas, due to the special molecular structure, LTN is more hydrophilic and polar in blood and tissues [85]. Therefore, it has distinctive localization in the cell membrane, optimizing its contact with highly oxidizable lipids and maximizing its protection against oxidative damage [85,86]. Regarding the improvement effect of co-supplementation with LTN and BS, which reported the best antioxidant status for all studied parameters, this finding was similar to the results of a study on black seabream, *Acanthopagrus Schlegelii,* fed L-carnitine and BS, which showed higher CAT activity when compared to fish fed individual supplementations [30]. The improvements associated with the BL+LTN treatments could be attributed to the facilitation of LTN absorption and deposition in the fish body [63,78,79], which was confirmed by the current findings of skin, muscle, and whole-body carotenoid content (Table 4).

The integrity examination of liver in the present study showed an improvement of hepatocyte function, as indicated in AST, ALT, and triglyceride levels in fish fed the BS and/or LTN supplemented diets. ALT and AST activities are a clinical diagnostic tool for liver injury evaluation [30,87]. In accordance with the present findings, LTN supplementation reduced plasma ALT and improved hepatic architecture in guinea pigs [78]. In addition, rainbow trout, *Oncorhynchus mykiss,* fed an LTN supplemented diet showed lower ALT and AST activities and lipid peroxide level in the liver [19]. These improvements of liver function could occur because the liver is the major storage organ for LTN and other carotenoid sources [78]. In addition. Lutin supplementation reduced serum total cholesterol, triacylglycerol levels in Broiler Chickens [88]. 

Additionally, the black seabream, *A. schlegelii*, showed a reduction of hepatic triglyceride content and down regulation of pro-inflammatory cytokine with dietary BS and L-carnitine [30]. Broiler fed BS supplemented diets had a significant reduction in serum aminopeptidases [87].

Furthermore, lipase activity was improved significantly with different supplemented groups (BS and/or LTN). In accordance, turbot, *Scophthalmus maximus,* fed a BS supplemented diet had improved lipase activity in the digesta and modulated whole body lipid content and lipid digestibility [89]. Additionally, the intestinal lipase and lipoprotein lipase activities were enhanced with BS supplementation [87]. The present findings could reflect an improvement of lipid utilization (digestion and absorption), as indicated in increasing whole-body lipids in BS supplemented treatments. Moreover, the improvement of lipase activity in hepatopancreas homogenates with LTN could also indicate better hepatopancreas function, and thus it could be related to the improvement of the inflammation response (decreasing pro-inflammatory, oxidative stress, and increasing anti-inflammatory) with LTN supplementation [81].

## 5. Conclusions

The present study revealed the synergistic effects of co-dietary supplementation of LTN and BS in improving growth performance, feed efficiency, and survival of the rabbitfish, *S. rivulatus* larvae. In addition, the carotenoid content of skin, muscle, and the whole body was improved with LTN supplementation and maximized with LTN and BS supplementation. The antioxidant balance (higher T-AOC, GSH-Px, and CAT activities, and lower MDA levels) improved in all supplemented groups, especially in LTN and BS+LTN groups. Also, BS supplementation modulated body lipid content and triglyceride and cholesterol levels and stimulated lipase activity in rabbitfish, *S. rivulatus*, supplemented groups. Further studies could be conducted to improve LTN absorption and deposition in fish larvae, such as nano-emulsification. In addition, its effect in large rabbit fish need to be validated.

## Figures and Tables

**Table 1:** Ingredients and proximate chemical composition of the experimental diet (g kg^−1^; on dry matter basis).

Ingredients	Diets (g kg^−1^)			
	Control	BS	LTN	BS+LTN
Soybean seed meal, 48%	362.00	362.00	362.00	362.00
Fishmeal anchovy, 65%	70.00	70.00	70.00	70.00
Sunflower seed meal	70.00	70.00	70.00	70.00
Soya protein concentrate	87.00	87.00	87.00	87.00
Whole wheat flour	190.00	190.00	190.00	190.00
Corn starch	107.00	106.85	106.90	106.75
Sunflower oil	80.00	80.00	80.00	80.00
Binder (Carboxy methyl cellulose)	10.00	10.00	10.00	10.00
Vitamin premix ^a^	10.00	10.00	10.00	10.00
Mineral premix ^b^	5.00	5.00	5.00	5.00
Monocalcium phosphate	8.00	8.00	8.00	8.00
Attractant (1 glycine and 2 betaine)	1.00	1.00	1.00	1.00
Bile salt (BS) ^c^	-	0.15	-	0.15
Lutein (LTN) ^d^	-	-	0.10	0.10
Proximate analysis (g kg^−1^)				
Dry matter (DM)	875.10	874.30	866.30	879.00
Crude protein (CP)	343.70	349.00	334.80	345.80
Ether extract (EE)	98.10	96.20	99.60	93.10
Ash	58.50	58.10	61.20	55.80
Nitrogen free extract (NFE) ^e^	499.70	496.70	504.40	505.30
Gross energy (GE; kJ g^−1^) ^f^	20.57	20.57	20.50	20.52

^a^ Vitamin premix (kg): VA 67 IU, VD 16.2 IU, VE 7.4 g, VK_3_ 340 mg, VB_1_ 670 mg, VB_2_ 1000 mg, VB_6_ 800 mg, VB_12_ 1.4 mg, VC 10 g, D-pantothenic acid 2.65 g, folic acid 330 mg, nicotinamide 5.35 g, choline chloride 35 g, biotin 34 mg, inositol 8 g. ^b^ Mineral premix (g/kg): FeSO_4_·H_2_O, 25.00 g; CuSO_4_·5H_2_O, 0.60 g; ZnSO_4_·H_2_O, 4.35 g; MnSO_4_·H_2_O, 2.04 g; KI, 1.10 g; NaSeO_3_, 2.50 g; MgSO_4_·H_2_O, 230.67 g. ^c^ Lutein powder (90–98%) used in this study were provided by Handan Qinyueming Metal Products Company Ltd. (Hebei, China). ^d^ The BS were a mixture of sodium glycocholate and sodium taurocholate and obtained from Jeevan Chemicals and Pharmaceuticals Co., (Maharashtra, India). ^e^ NFE calculated using the following equation: NFE = 1000 − (CP + EE + CF + ash) [47]. ^f^ GE calculated based on 23.6, 39.4 and 17.2 kJ GE g^−1^ protein, EE and carbohydrates, respectively.

**Table 2:** Effects of dietary supplementation with lutein (LTN) and bile salts (BS) on the growth performance, feed efficiency, and survival of *Siganus rivulatus* after 60 days of feeding.

	Diets			
	Control	BS	LTN	BS+LTN
Final body weight (g fish^−1^)	1.97 ± 0.02 ^b^	2.06 ± 0.07 ^b^	2.01 ± 0.01 ^b^	2.35 ± 0.03 ^a^
Weight gain (g fish^−1^)	1.79 ± 0.01 ^b^	1.88 ± 0.07 ^b^	1.83 ± 0.01 ^b^	2.18 ± 0.02 ^a^
Specific growth rate (% day^−1^)	3.94 ± 0.05 ^b^	4.01 ± 0.08 ^b^	4.02 ± 0.03 ^b^	4.37 ± 0.04 ^a^
Final body length (cm fish^−1^)	5.43 ± 0.03	5.47 ± 0.03	5.53 ± 0.07	5.83 ± 0.03
Body length growth rate (% day^−1^)	1.29 ± 0.04	1.32 ± 0.03	1.40 ± 0.06	1.40 ± 0.02
Feed efficiency (%)	35.76 ± 1.13 ^b^	38.60 ± 2.60 ^b^	37.08 ± 0.86 ^b^	46.55 ± 1.46 ^a^
Feeding rate (% BW day^−1^)	7.74 ± 0.23 ^ab^	7.27 ± 0.46 ^ab^	7.51 ± 0.16 ^a^	6.20 ± 0.22 ^b^
Condition factor	1.26 ± 0.02	1.23 ± 0.01	1.19 ± 0.04	1.19 ± 0.01
Survival (%)	86.67 ± 3.85 ^b^	91.11 ± 2.22 ^ab^	93.33 ± 3.85 ^ab^	97.78 ± 2.22 ^a^

Means in the same row with different superscripts are significantly (*p* < 0.05) different. Data are mean ± SE of three replicates.

**Table 3:** Effects of dietary supplementation with lutein (LTN) and bile salts (BS) on the whole-body composition (%; on wet weight basis) of *Siganus rivulatus* after 60 days of feeding.

Nutrient Component	Diets			
	Control	BS	LTN	BS+LTN
Moisture (%)	75.89 ± 0.48 ^a^	75.59 ± 0.28 ^ab^	75.64 ± 0.06 ^ab^	74.71 ± 0.28 ^b^
Crude protein (%)	14.61 ± 0.26 ^ab^	14.48 ± 0.06 ^b^	15.34 ± 0.04 ^ab^	15.63 ± 0.28 ^a^
Crude lipid (%)	4.97 ± 0.06 ^ab^	5.55 ± 0.0 ^ab^	4.81 ± 0.22 ^b^	5.59 ± 0.13 ^a^
Ash (%)	3.93 ± 0.41	4.43 ± 0.41	4.22 ± 0.28	3.97 ± 0.14

Means in the same row with different superscripts are significantly (*p* < 0.05) different. Data are mean ± SE of three replicates.

**Table 4:** Effects of dietary supplementation with lutein (LTN) and bile salts (BS) on the total carotenoid content (µg g^−1^) of feed and *Siganus rivulatus* after 60 days of feeding.

Carotenoids Content (µg g^−1^)	Diets			
	Control	BS	LTN	BS+LTN
Feed	13.88 ± 1.38 ^b^	14.51 ± 1.62 ^b^	107.55 ± 3.91 ^a^	110.75 ± 2.55 ^a^
Fish				
Skin	5.42 ± 0.22 ^b^	7.14 ± 0.70 ^b^	52.64 ± 3.72 ^a^	57.62 ± 2.70 ^a^
Muscle	0.20 ± 0.07 ^c^	0.54 ± 0.16 ^c^	8.01 ± 0.81 ^b^	13.43 ± 1.67 ^a^
Whole body	1.45 ± 0.05 ^c^	1.57 ± 0.20 ^c^	20.19 ± 2.47 ^b^	22.33 ± 3.79 ^a^

Means in the same row with different superscripts are significantly (*p* < 0.05) different. Data are mean ± SE of three replicates.
